# Nonlinear Optical Modulation Characteristics of MXene Cr_2_C for 2 μm Pulsed Lasers

**DOI:** 10.3390/nano13131965

**Published:** 2023-06-28

**Authors:** Jingcheng Yu, Zijun Chen, Tao Li, Tianli Feng, Jiacheng Huang, Yizhou Liu, Zheng Ni, Li Yu, Wenchao Qiao

**Affiliations:** 1 School of Information Science and Engineering, Shandong University, Qingdao 266237, China; 2 Shandong Provincial Key Laboratory of Laser Technology and Application, Shandong University, Qingdao 266237, China; 3 China Key Laboratory of Laser & Infrared System (Ministry of Education), Shandong University, Qingdao 266237, China; 4 CHN Energy Shouguang Company, Weifang 262714, China; 5 Qingdao Institute of Measurement Technology, Qingdao 266000, China

**Keywords:** passively Q-switched, Tm: YAP solid laser, saturable absorber, Cr_2_C MXene

## Abstract

MXene materials have shown numerous useful mechanical and electronic properties, and have been found to possess nice potential in the field of optical modulation. Here, we fabricated a MXene Cr_2_C saturable absorber by the liquid-phase exfoliation method, and systemically analyzed the surface morphology and nonlinear properties of the Cr_2_C sample. Applying the Cr_2_C saturable absorber as a Q-switch in a thulium-doped yttrium aluminum perovskite (Tm: YAP) laser, the shortest single pulse was obtained with a width of 602 ns under an absorbed pump power of 3.3 W at a repetition rate of 55 kHz with a T = 1% output coupler. The maximum output power was obtained with a T = 5% output coupler at a repetition rate of 58 kHz. The obtained maximum pulse energy and peak power were 3.96 μJ and 4.36 W, separately, which reveal that the MXene Cr_2_C can be applied as a promising modulation material in the near-infrared pulsed lasers.

## 1. Introduction

In recent years, nanomaterials have been widely applied in medicine, energy, microelectronics and other fields due to their magnetic, electrical, optical and chemical proper-ties [1]. For example, Abouzar et al. investigated nanomaterials-based capacitors and applied them in the field of deoxyribonucleic acid (DNA) detection [2]; Schöning et al. reported the latest technologies and development trend advancements of biosensitive field effect devices (FED) with applied nanomaterials [3]. As a novel two-dimensional nanomaterial, MXene has attracted the attention of many researchers since it was firstly reported by Naguib et al. in 2011 [4]. MXenes have been applied in the field of energy storage and conversion [5,6], field-effect transistor (FET) [7,8], catalysis [9,10,11] and sensors [12,13,14] because of their excellent electronic and optical properties. Moreover, in the field of optical modulation, the 2D nanomaterials MXenes have been demonstrated as promising saturable absorbers (SAs) candidates due to their remarkable nonlinear modulation properties [15]. In the last few years, Wang et al. conducted studies on the nonlinear optical properties of MXene Mo_2_C, and successfully employed it as a SA for passively Q-switched lasers at 1 and 1.3 μm due to its high modulation depth [16,17]. Recently, a novel MXene material called Cr_2_C has attracted much attention in the fields of hydrogen storage [18] and electrical conduction [19]. Due to its similar structure but more stable physical and chemical properties compared to Mo_2_C, Cr_2_C is reasonably expected to possess similar or even better optical modulation and other optical responses. However, there is still no relevant report for the optical modulations of Cr_2_C MXene in the generation of pulsed lasers, leaving an unexplored territory of this material.

Based on the physical and chemical properties of Cr_2_C, we expect to apply Cr_2_C materials in the modulation field of 2 μm pulsed lasers. The eye safety lasers operating at a 2 μm laser have significant applications in atmospheric remote sensing [20], coherent LIDAR [21,22], industrial material processing [23] and medical scalpels in surgery [24]. Compared with continuous wave (CW) lasers, pulsed lasers with a high peak power and large pulse energy are more addictive in laser surgery and industrial processing. To achieve the pulsed laser output, one of the most efficient solutions is Q-switching. By employing various novel saturable absorption materials, such as graphene, black phosphorus, transition metal oxides and topological insulators, passive Q-switched lasers (PQS) can be achieved economically and compactly.

**Figure 1:**
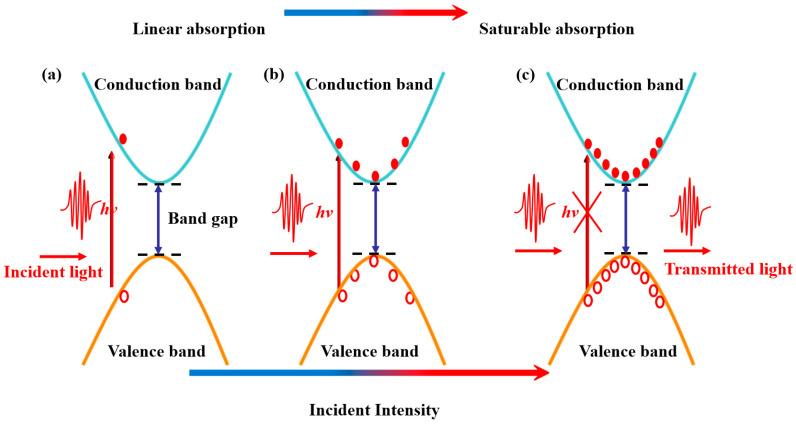
Schematic diagram of nonlinear saturable absorption process. (**a**) Photoexcitation electron transition, (**b**) carrier thermal equilibrium and (**c**) absorption blocking.

The saturable absorption of materials refers to the nonlinear optical phenomenon caused by the Pauli blocking effect which occurs under high intensity light [25]. When a beam of light with photon energies is larger than the band gap of the nonlinear optical material, the electrons in the valence band of the material will transit to the conduction band under the excitation of the incident photons. As shown in Figure 1a, at low incident light intensity, most of the photons are absorbed by the material, which is called the linear absorption state, exhibiting low transmittance. As the incident light intensity increases, more electrons in the valence band are excited to the conduction band until the electrons in the valence band are depleted and the conduction band is occupied by the carrier, as shown in Figure 1c. Thus, most incident photons cannot be absorbed and the material exhibits high transmittance in the saturable absorption state [26].

In this paper, we systematically investigated the optical response of MXene Cr_2_C materials and focused on the nonlinear optical modulation properties of MXene Cr_2_C as a saturable absorber for a 2 μm pulsed laser. Firstly, Cr_2_C MXene SA was successfully prepared by liquid-phase exfoliation (LPE), and its morphology was characterized in detail by X-ray diffraction (XRD) and atomic force microscopy (AFM) in the following experiment. Then, the transmittance spectral and nonlinear optical responses of Cr_2_C SA were measured by the UV-Vis-NIR spectrophotometer and open-aperture Z-scan method, respectively. Finally, we achieved a stable passively Q-switched Tm: YAP laser operation at 1985 nm employing the Cr_2_C SA. Under an absorbed pump power of 3.3 W, the maximum output power of 221 mW was obtained with a T = 5% output coupler (OC), corresponding to a slope efficiency of 17.6%. The shortest pulse duration was measured to be 602 ns with a repetition rate of 55 kHz with a T = 1% OC. The experimental results demonstrate the potential applications of MXene Cr_2_C in the field of nonlinear optical modulation.

## 2. Preparation and Characterization

The Cr_2_C SA was fabricated by the conventional ultrasound-assisted LPE technique. During the preparation of SA, 5 mg of Cr_2_C powder was firstly dissolved in 5 mL of anhydrous isopropyl alcohol solution. After ultrasonic shaking for 2 h, the Cr_2_C dispersion was further centrifuged at 3000 rpm for 15 min by the differential centrifugation method. Then, the supernatant liquid was collected and dropped onto a quartz substrate with a size of 2 × 2 × 0.5 mm³. Subsequently, the quartz substrate was placed on a rotating coater and coated for 1 min at a speed of 500 rpm. Finally, the Cr_2_C SA was prepared after an air-drying treatment at a normal atmospheric temperature.

**Figure 2:**
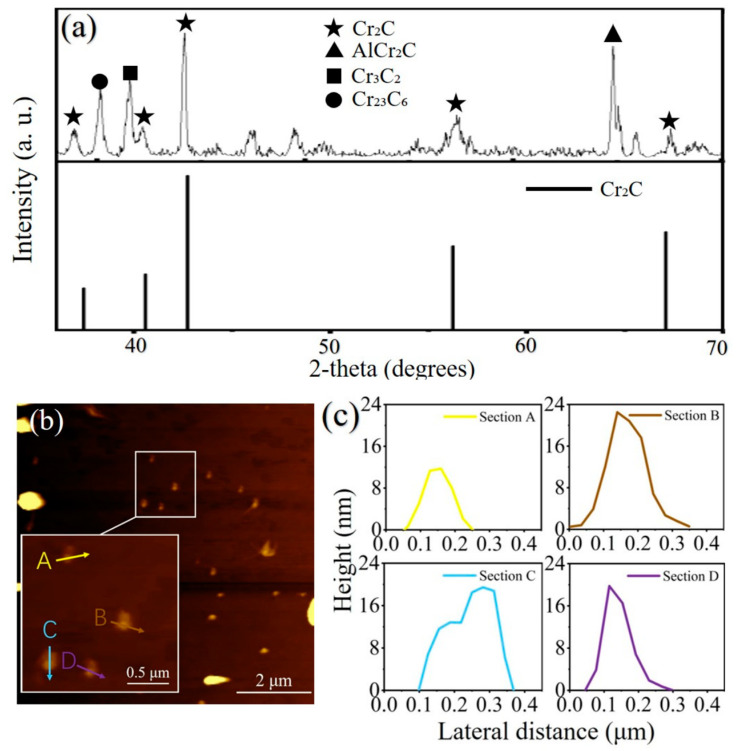
The characterization of the Cr_2_C sample. (**a**) The powder X-ray diffraction of Cr_2_C MXene SA. (**b**) The AFM image. (**c**) The height profile of the Cr_2_C nanosheet.

To confirm the constituent of the Cr_2_C MXene sample, X-ray diffraction (Bruker AXS D8 Advance, Karlsruhe, Germany) measurement was performed, and the results were shown in Figure 2a. There are mainly four kinds of materials in the sample: the Cr_2_C (black star), the AlCr_2_C (black triangle), the Cr_3_C_2_ (black square) and the Cr_23_C_6_ (black circle). As the Cr_2_C MXene materials were fabricated by selectively etching Al atoms from the MAX phase of AlCr_2_C, the AlCr_2_C detected by XRD should be the residue from the etching process [27]. For the Cr_3_C_2_ and the Cr_23_C_6_, they would both be the by-products of the deterioration of the Cr_2_C MXenes [28]. Compared with the standard Cr_2_C structure data (ICDD database no. PDF 14-0519) [29], it can be found that the content of Cr_2_C in the sample is predominant, which indicates that the properties presented by the sample in the experiment should be mainly derived from the Cr_2_C material. Furthermore, the irregularity of the baseline may be caused by the unsatisfactory crystallinity of the sample and some amorphous materials.

To visualize the morphological features of Cr_2_C SA, the atomic force microscopy (AFM; HORIBA SmartSPM, Kyoto, Japan) measurement was employed. The AFM image of the Cr_2_C MXene sample and the typical height profile diagrams shown in Figure 2b,c clearly indicated that the Cr_2_C MXene was exfoliated successfully into a 2D layered sheet with a nanoscale size. As shown in Figure 2b, Cr_2_C was extensively and quasi-uniformly attached to the substrate after preparation by liquid-phase stripping and the size of the material in the 2D plane was on the order of micrometers. There are several sparse bright spots at the edge of Figure 2b, which refer to residual bulk particles due to incomplete stripping. These bulk particles do not substantially interfere with the subsequent experimental results because they are sparsely distributed. The height profiles of the Cr_2_C nanosheets measured along the A, B, C and D lines are shown in Figure 2c, reflecting the thickness distribution of the Cr_2_C nanosheets between 10 nm and 25 nm. These results indicate that we successfully prepared Cr_2_C materials into Cr_2_C nanosheets by the liquid-phase exfoliation (LPE) method.

**Figure 3:**
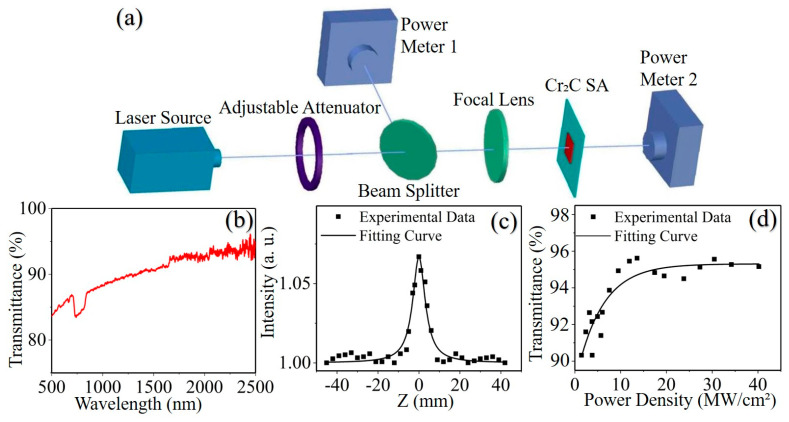
(**a**) Schematic diagram of the open-aperture (OA) Z-scan. (**b**) Linear optical transmittance spectrum of Cr_2_C SA. (**c**) OA Z-scan results. (**d**) Nonlinear transmittance curve.

The transmittance characteristics of Cr_2_C SA were further confirmed by measuring the optical transmittance spectrum of Cr_2_C SA between 500 and 2500 nm using a UV-Vis-NIR spectrophotometer (Hitachi UH4150, Tokyo, Japan). As shown in Figure 3b, the transmittance of the Cr_2_C SA was higher than 80% in the whole spectrum and above 92% near the 2 μm region, which indicates that Cr_2_C SA has a certain absorption capacity and broadband transmittance characteristics. Moreover, since one lamp can hardly cover the entire wavelength range, we switched the lamp during the course of the experiment, which resulted in two obvious fluctuations in the transmission curve at wavelengths of 660 nm and 1650 nm.

To probe the nonlinear optical properties of Cr_2_C SA at 2 μm, we performed Z-scan measurements with a homemade OA Z-scanner, employing a 2 μm laser with 800 Hz repetition rate and 100 ns pulse width. As shown in Figure 3a, to improve the operability of the experiment and the reliability of the results, the Cr_2_C MXene sample was mounted on the guide rail between the focal lens and the power meter, so that the sample can be moved along the guide rail. Based on this design, we can move the SA on the guide rail to change the incident saturation fluence on the sample during the experiment. As illustrated in Figure 3c, the normalized transmittance curve gradually reached the maximum with the Cr_2_C SA approaching the focus position symmetrically, indicating the expected nonlinear absorption response of the as-prepared Cr_2_C at 2 μm.

In order to quantify the nonlinear absorption capability of the as-prepared Cr_2_C, we numerically fitted the Z-scan experimental data by the formula [30]:

(1) $$T=\sum_{m=0}^{\infty }\frac{{\left[-q_{0}\left(z,0\right)\right]}^{m}}{{\left(m+1\right)}^{1.5}},\;m\in N,$$

(2) $$q_{0}(z,0)=\frac{\beta_{eff}L_{eff}I_{0}}{\left(1+z^{2}/{z_{0}}^{2}\right)},$$

where $L_{eff}=(1-e^{{L\alpha }_{0}})/\alpha_{0}$ is the effective thickness of the nonlinear optical material, *α_0_* is the linear absorption coefficient, *L* is the actual thickness of the sample, *I_0_* represents the maximum optical power density at the focus and *β_eff_* represents the effective nonlinear absorption coefficient of the material. By fitting the measured Z-scan results, the *β_eff_* was calculated to be −(2.75 ± 0.08) × 10^−2^ cm/GW.

To further confirm the nonlinear saturable absorption properties of the as-prepared Cr_2_C, we performed the I-scan measurement by adding an adjustable light attenuator to the Z-scan setup. The transmittance of the sample versus the beam intensity was measured by varying the incident beam intensity with an adjustable attenuator while the position of the sample was fixed. The nonlinear transmission curve was obtained by fitting the I-scan experimental data with the following formula [30]:

(3) $$T=1-∆Texp\left(-I/Is\right)-T_{ns},$$

where Δ*T* is the modulation depth, representing the maximum amount of variation in the material transmission rate, which affects the width of the output pulse. *I* represents the input intensity. $I_{S}=hv/2\sigma_{A}\tau_{A}$ is the saturation intensity, in which *hv* is the photon energy, *σ_A_* is the absorption cross section of the material and *τ_A_* represents the saturation recovery time required for the material from the bleached state to the linear absorption state, which is the decisive factor of the narrowest pulse width. The non-saturable losses *T_ns_* refers to the scattering loss or Fresnel loss due to the roughness of the material surface. It should be pointed out that excessive non-saturable losses will suppress the operation efficiency of the laser.

As shown in Figure 3d, the nonlinear transmittance increased with the power density and gradually reached saturation. The fitted curve gave the modulation depth *ΔT*, the saturation intensity *I_S_* and the non-saturable losses *T_ns_* of the Cr_2_C SA as 7.5%, 6.7 MW/cm^2^ and 4.35%, respectively. It follows that Cr_2_C SA has excellent nonlinear optical properties, which indicates its potential as a candidate for laser pulse modulation.

## 3. Results and Discussion

**Figure 4:**
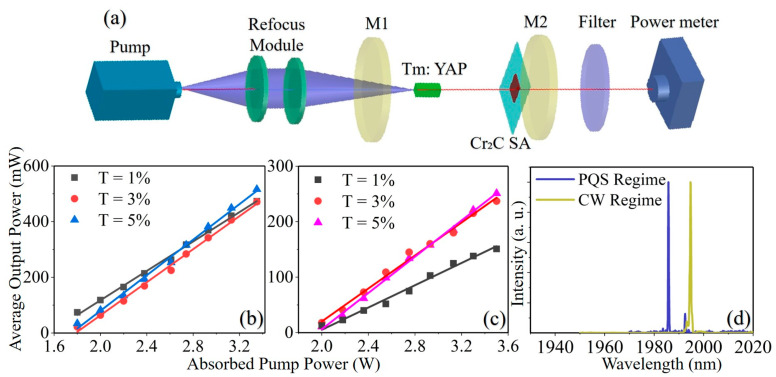
(**a**) Schematic diagram of the Cr_2_C Q-switched Tm: YAP laser. The average output power of (**b**) CW and (**c**) pulsed Cr_2_C lasers versus absorbed pump power. (**d**) The emission spectra of the Tm: YAP laser.

To further confirm the saturation absorption properties of the prepared Cr_2_C SA, a Q-switched Tm: YAP laser was designed employing the Cr_2_C SA, and the diagram of the laser device was shown in Figure 4a. In the device, an 18 mm plane-plane straight cavity was built consisting of an input coupler (M1) and an output coupler (M2). The M1 was coated with anti-reflectivity (AR) and high-reflection (HR) at 790 nm and 1940 nm, separately. We successively utilized three flat mirrors with a different transmittance at 2 μm as the output couplers during the measurements, and the transmittance of the OC was 1%, 3% and 5%, respectively. The gain medium was a (3 × 3 × 10) mm^3^ size a-cut Tm: YAP crystal (doping concentration: 3 at%) with large thermal conductivity. The thermal load in the laser operation can be eliminated by mounting the gain medium wrapped with indium foil onto a water-cooled copper radiator cooled at 15 °C. The 794 nm pump light was generated by a laser diode and guided by an optical fiber with a core diameter of 400 µm and a numerical aperture of 0.22. To focus the pump beam on the gain medium properly, we employed an optical refocus module (1:1) consisting of two focus lenses. A filter was installed at the back of the M2 to eliminate the residual pump energy. A power meter (Thorlabs PM100D, Newtown, NJ, USA) was utilized to measure the output power, while a digital fluorescence oscilloscope (Tektronix DPO42102B-L, Beaverton, OR, USA) was connected to an InGaAs PIN photodetector (EOT ET-5000, MI, USA) to keep a record of the pulse temporal behavior.

We firstly performed the continuous wave (CW) laser operation without the Cr_2_C SA. Figure 4b shows that the CW Tm: YAP laser average output powers increased versus the absorbed pump powers with three different OCs of T = 1%, 3% and 5%, respectively. As the absorption pump power continued to increase, the average output power of the CW Tm: YAP laser increased roughly linearly. Under the absorbed pump power of 3.3 W, the maximum output power of 516 mW was obtained with a T = 5% OC, corresponding to a slope efficiency of 31.1%. In addition, a stable passive Q-switched (PQS) laser operation was achieved by inserting Cr_2_C SA into the cavity near the OC. The variation trend of the output power of a Q-switched Tm: YAP laser was demonstrated in Figure 4c. The obtained maximum average output power was 221 mW with the T = 5% OC under an absorbed pump power of 3.3 W, with a slope efficiency of 17.6%. Comparing the operating characteristics of CW and Q-switched lasers, the pumping threshold of the Q-switched regime was higher than that of the CW regime, while the average output power was lower, which was due to the increased loss after the insertion of Cr_2_C SA. The instability of the Q-switched laser operation increased when the absorbed pump power exceeded 3.3 W, probably due to the limitation of the thermal effects as well as the possible oversaturation of the Cr_2_C SA.

As shown in Figure 4d, the emission spectra of the Tm: YAP laser operating in the CW and Q-switched regimes with the OC of T = 1% were measured by a laser spectrometer (APE Wave Scan, Berlin, Germany). The center wavelength was 1995.4 nm when the laser operating in the CW regime. After inserting Cr_2_C SA into the cavity, the output wavelength blue-shifted to 1985 nm, which was mainly caused by the insertion loss of the saturable absorber.

**Figure 5:**
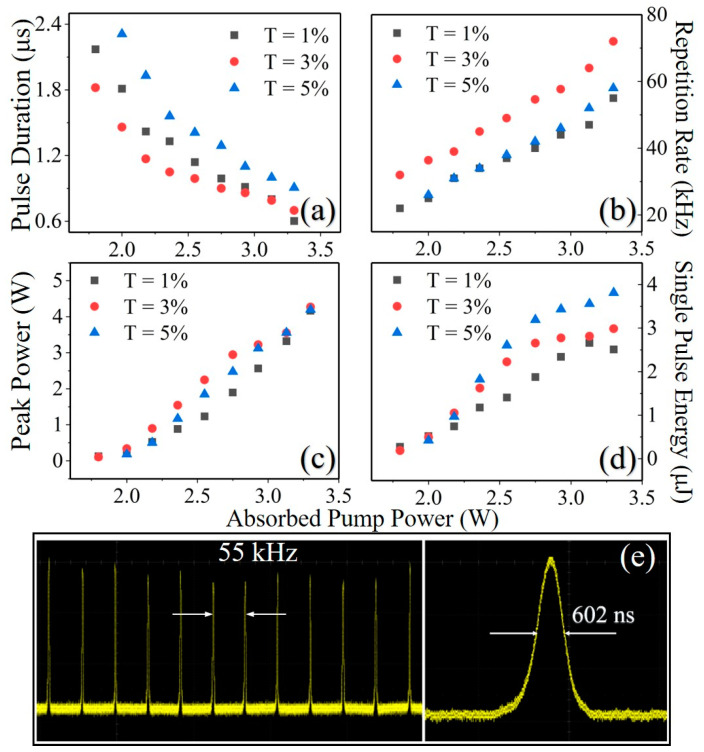
(**a**) Pulse duration. (**b**) Pulse repetition rate. (**c**) Peak power. (**d**) Single-pulse energy of PQS laser versus absorbed pump power. (**e**) Typical pulse train and temporal pulse profile.

To further verify the nonlinear optical modulation performance of the Cr_2_C SA, we measured the pulse duration and repetition frequency of the PQS laser, which are shown in Figure 5a,b. The pulse duration continuously decreased with the increasing absorbed pump power. Under the pump power of 3.3 W, the shortest pulse duration of 601 ns, 699 ns and 907 ns were obtained with OCs of T = 1%, 3% and 5%, respectively. In contrast to the variation trend of the pulse durations, the repetition rates increased with the absorbed pump power. As shown in Figure 5b, the repetition rates of the lasers under different OCs increased from 22 kHz, 32 kHz and 26 kHz to 55 kHz, 72 kHz and 58 kHz, respectively. Figure 5c shows the peak powers increased with the absorbed pump power. Among the three lasers, the obtained maximum peak powers were 4.16 W, 4.27 W and 4.36 W with a transmittance of T = 1%, 3% and 5%, respectively. The dependences of single pulse energy on the absorbed pump power were shown in Figure 5d. With the increase of pump power, the single-pulse energy increased almost linearly at the beginning and gradually reached saturation, which could be attributed to the thermal effect of the laser crystal and the oversaturation of the SA. The maximum single-pulse energies for the OCs of T = 1%, 3% and 5% were 2.51 μJ, 2.99 μJ and 3.96 μJ, respectively. A typical pulse train at T = 1% and a temporal pulse profile with a pulse duration of 602 ns were recorded and shown in Figure 5e. During a two-hour detection period, the pulsed laser maintained a stable output and the maximum output power instability was less than 7%, indicating the long-term stability of Cr_2_C SA. All of these experimental results demonstrated the stable Q-switched performance and the potential optical modulation applications for the Cr_2_C SA.

Table 1 shows the PQS solid-state lasers with other 2D nanomaterials as SAs at 2 μm, including Mo_2_C [16], BP [31], graphene [32], MoS_2_ [33], WS_2_ [33], MoTe_2_ [34] and Mg-MOF-74 [35]. Compared to the other 2D nanomaterials, Cr_2_C has unique balanced advantages at optical modulation.

## 4. Conclusions

In this work, we present the first demonstration of a passive Cr_2_C Q-switched stable 2 μm laser. A Cr_2_C saturable absorber was successfully fabricated by a liquid-phase exfoliation technique. The morphology and the nonlinear optical properties of Cr_2_C SA were comprehensively investigated. Through Z-scan experiments and I-scan experiments, the effective nonlinear absorption coefficient, the modulation depth, the saturation intensity and the non-saturable losses of Cr_2_C SA were obtained with −(2.75 ± 0.08) × 10^−2^ cm/GW, 7.5%, 6.7 MW/cm^2^ and 4.35%, respectively, indicating the potential of Cr_2_C SA for pulsed laser modulation. Under a pump power of 3.3 W, we obtained the maximum average output power of 221 mW at a repetition rate of 58 kHz with an OC of T = 5%, corresponding to the maximum single-pulse energy of 3.96 μJ and peak power of 4.36 W. The shortest pulse duration was 602 ns at a repetition rate of 55 kHz with a T = 1% OC. By applying a T = 3% OC, the repetition rates of the laser pulses can reach 72 kHz. Compared to the other 2D nanomaterials, Cr_2_C has unique balanced advantages at optical modulation. These results validate that MXene Cr_2_C materials have effective nonlinear absorption properties and modulation capabilities. This work is of significance in the laser pulse generation for future research, especially in the modification of novel low-dimensional nanomaterials of nonlinear saturable absorption properties, and is also profound for expanding the application field of Cr_2_C materials.

## Figures and Tables

**Table 1:** The PQS solid-state lasers characters with 2D nanomaterials as SAs at 2 μm.

Materials	Output Power (mW)	Pulse Duration (ns)	Pulse Repetition Rate (kHz)	Single Pulse Energy (μJ)	Peak Power (W)	Crystal	Reference
Mo_2_C	547	136	261	2.09	15.41	Nd: YAG	[16]
black phosphorus	151	1780	19.25	7.84	4.4	Tm, Ho: YAP	[31]
graphene	310	285	190	5.61	1.6	Tm: KLu(WO_4_)_2_	[32]
MoS_2_	410	458.8	83.1	4.93	10.7	Tm: YAP	[33]
WS_2_	668	528.4	87.7	7.62	14.4	Tm: YAP	[33]
MoTe_2_	1210	380	144	8.4	22.2	Tm: YAP	[34]
Mg-MOF-74	660	313	117	5.6	18	Tm: YAP	[35]
Cr_2_C	155	602	55	3.96	4.36	Tm: YAP	this work

## Data Availability

Data will be made available on request.
